# Development and Validation of a Prognostic Gene Signature in Clear Cell Renal Cell Carcinoma

**DOI:** 10.3389/fmolb.2021.609865

**Published:** 2021-04-08

**Authors:** Chuanchuan Zhan, Zichu Wang, Chao Xu, Xiao Huang, Junzhou Su, Bisheng Chen, Mingshan Wang, Zhihong Qi, Peiming Bai

**Affiliations:** ^1^ Shaoxing people’s Hospital, Shaoxing, China; ^2^ Zhongshan Hospital, Xiamen University, Xiamen, China; ^3^ Nanchang Five Elements Bio-Technology Co., Ltd, Nanchang, China

**Keywords:** kidney cancer, microarray, WGCNA, targeting therapy, novel markers, prognostic model

## Abstract

Clear cell renal cell carcinoma (ccRCC), one of the most common urologic cancer types, has a relatively good prognosis. However, clinical diagnoses are mostly done during the medium or late stages, when mortality and recurrence rates are quite high. Therefore, it is important to perform real-time information tracking and dynamic prognosis analysis for these patients. We downloaded the RNA-seq data and corresponding clinical information of ccRCC from The Cancer Genome Atlas (TCGA) and Gene Expression Omnibus (GEO) databases. A total of 3,238 differentially expressed genes were identified between normal and ccRCC tissues. Through a series of Weighted Gene Co-expression Network, overall survival, immunohistochemical and the least absolute shrinkage selection operator (LASSO) analyses, seven prognosis-associated genes (AURKB, FOXM1, PTTG1, TOP2A, TACC3, CCNA2, and MELK) were screened. Their risk score signature was then constructed. Survival analysis showed that high-risk scores exhibited significantly worse overall survival outcomes than low-risk patients. Accuracy of this prognostic signature was confirmed by the receiver operating characteristic curve and was further validated using another cohort. Gene set enrichment analysis showed that some cancer-associated phenotypes were significantly prevalent in the high-risk group. Overall, these findings prove that this risk model can potentially improve individualized diagnostic and therapeutic strategies.

## Introduction

In 2019, an estimated 73,820 patients were diagnosed with renal cell cancer, with a mortality burden of 14,000 persons, indicating a high mortality rate from this disease (SEER http://seer.cancer.gov/statfacts/html/kidrp.html). Clear cell renal cell cancer is the most common and lethal subtype of renal carcinoma, accounting for approximately 75% of kidney cancer (Moch et al., 2016). Currently, surgical therapy has been shown to be effective in the treatment of localized renal cell carcinoma. However, the medium or late stage diagnoses of this cancer have been associated with high mortality and recurrence rates. The tyrosine kinase inhibitor (TKI) and mammalian target of rapamycin (mTOR) inhibitors have improved therapeutic outcomes. To a certain extent, most patients develop resistance or discontinue the use of these drugs due to severe side effects (Banumathy and Cairns, 2010; Suttle et al., 2014; Lai et al., 2016). Therefore, to improve the quality of life for these patients, it is important to perform real-time information tracking and dynamic prognostic analyses.

Due to advances in microarray and high throughput technologies, several candidate biomarkers associated with ccRCC have been identified using bioinformatics analysis (Sun et al., 2019; Yan et al., 2019). Unfortunately, most studies did not evaluate the correlation between genes and clinical characteristics. The weighted gene co-expression network analysis (WGCNA), characterized by the presence of different genes with similar expression patterns in the same module, has been used to determine the relationships between module and clinical traits. Recently, it has been used to screen candidate biomarkers for complex diseases, including (Voineagu et al., 2011), Alzheimers (Miller et al., 2010) and glioblastoma (Horvath et al., 2006).

In this study, we identified multiple differentially expressed genes associated with KIRC using high-throughput bioinformatics analysis of data obtained from the Gene Expression Omnibus database. Subsequently, we used WGCNA to select a clinically significant module. Furthermore, screening was done to identify the real hub genes. Using the real hub genes, we constructed and validated a prognostic multigene signature using the cancer genome atlas cohort. Finally, functional enrichment analysis was performed to determine the underlying mechanisms.

## Materials and Methods

### Research Design and Data Collection

**FIGURE 1:**
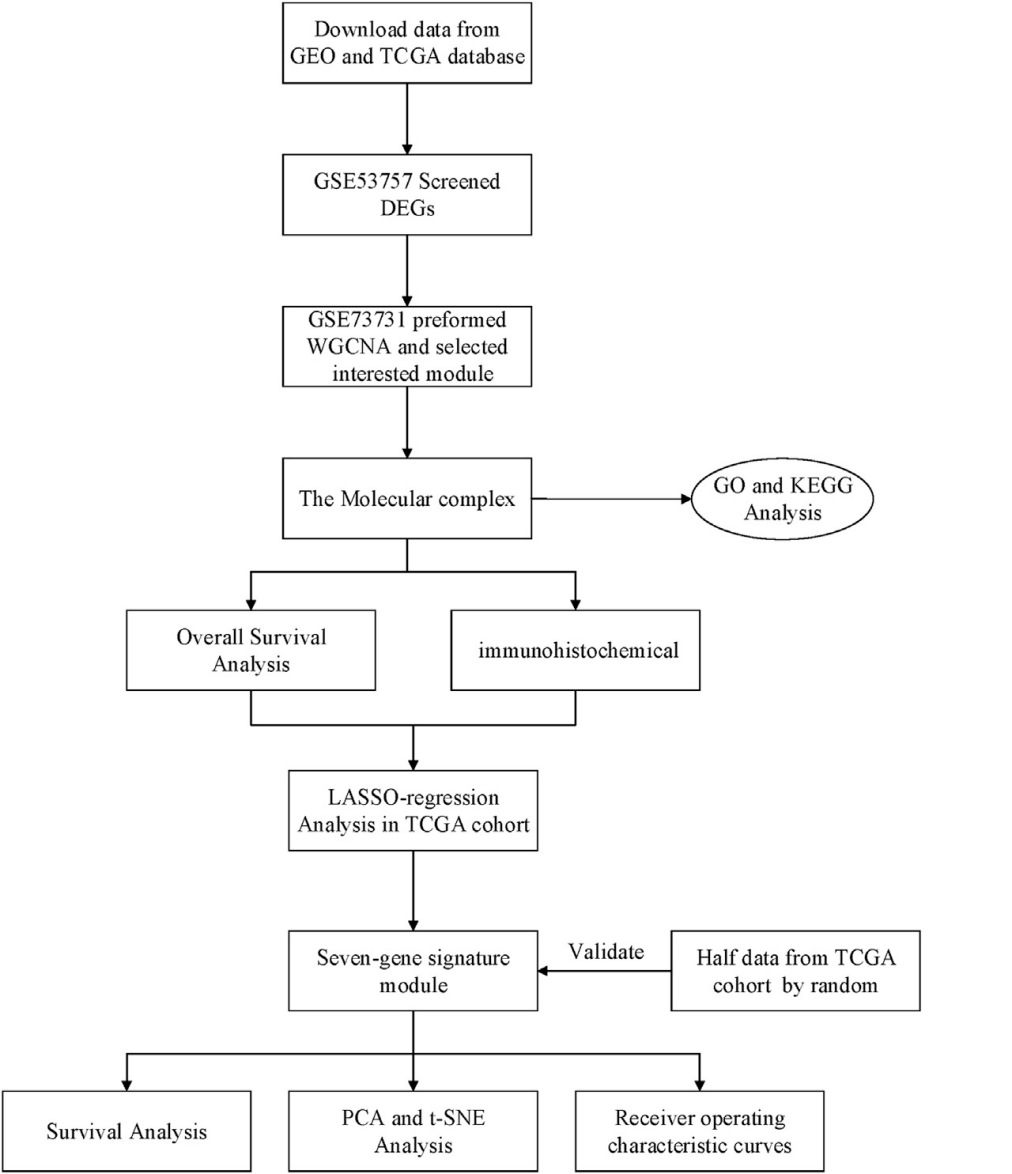
Flow chart of data collection and analysis.

Raw gene expression profiles and clinical data were obtained from the Gene Expression Omnibus (GEO) database (https://www.ncbi.nlm.nih.gov/geo/) (Table 1). Dataset GSE53757, including 144 samples (72 normal kidney tissue, 72 kidney renal cell carcinoma) was used to screen for the differently expressed genes (DEGs). Dataset GSE73731 had 265 samples, however, most of them did not have their clinical data. Therefore, 125 samples from the GSE73731 dataset were finally used to identify the hub module through WGCNA. The TCGA data was used to construct and validate the prognostic risk model. Further, we used GSE89563, an independent dataset, to perform Gene Set Enrichment Analysis (GSEA). The data collection and analysis procedures was as shown in Figure 1.

**TABLE 1:** Detailed information about datasets.

Datasets	GSE53757	GSE73731	GSE89563	TCGA and GTEx
Platform	HGU133_Plus_2	HGU133_Plus_2	HuGene–2_1–st	Illumin
	GPL570	GPL570	GPL17692	RNAseqV2
Sample number				
Total	144	125	16	623
Normal kidney	72	-	-	100
Kidney cancer	72	125	16	523
Tumor stage				
Stage i	24	41	5	265
Stage ii	19	12	3	57
Stage iii	14	28	4	123
Stage iv	15	44	4	82
unknown	-	-	-	3
Pathology grader				
Grade i	-	17		
Grade ii	-	45		
Grade iii	-	44		
Grade iv	-	76		
Function	Select DEGs	Perform WGCNA	Perform GSEA	Related verification

### Data Processing and Screening for Differentially Expressed Genes

Raw microarray data were subjected to RMA background correction, log2 transformation and normalized by quantile normalization. The “Affy” R packages were used to summarize the Median-polish probe sets (Gautier et al., 2004). The Affymetrix annotation files were used to annotate probes. The assessment of microarray quality was performed by sample clustering based on the distance between different samples in Pearson’s correlation matrices and average linkage. Then, the R package “limma” (Ritchie et al., 2015) was used to select the DEGs.

### Weighted Gene Co-expression Network Construction

Using the R package “WGCNA,” the DEGs were used to construct a weighted co-expression network (Zhang and Horvath, 2005). First, the “goodSamplesGenes” R package in the “WGCNA” packages was used to determine whether the input DEGS were good genes from good samples. Second, we constructed an adjacent matrix by Pearson’s correlation analysis of all gene pairs. To construct a scale-free co-expression network, we used a soft-thresholding parameter (β), which could enhance the strong correlations between genes and penalize weak correlations. The adjacency matrix was then turned into a topological overlap matrix (TOM). The TOM was used to measure network connectivity of a gene, which was defined as the sum of its adjacency with all other genes and was used for network generation. Finally, based on TOM dissimilarity, we performed the average linkage hierarchical clustering. The purpose of this step was to classify genes with similar expression patterns into gene modules with a minimum size of 50.

### Identification of Clinically Significant Modules and Module Functional Annotation

After the classification of differentially expressed genes into gene modules, which were characterized by similar expression patterns, WGCNA was used to determine the correlation between the external clinical information and gene modules to identify clinically significant gene modules. Combined with the correlative clinical feature, the gene module that was most correlated with clinical features was selected as the hub module.

### Screening Tests

Based on the previous step, hub genes were input into the STRING (https://string-db.org/) database to construct a protein-protein interaction (PPI) network. The minimum interaction score was >0.4. The Cytoscape software (Su et al., 2014) and Molecular Complex Detection tool (MCODE) (version 1.5.1) (Bader and Hogue, 2003), a cytoscape plug-in, were used to visualize and identify the most significant module in the PPI network. The resulting criteria were: cluster finding = haircut, cut-off degree = 2, cut-off node score = 0.2, k-score = 2, and maximum depth = 100. We used the Gene Expression Profiling Interactive Analysis (GEPIA) database (http://gepia.cancer-pku.cn/), with data obtained from the TCGA and GTEx database to test the diagnostic and survival-related value of hub genes. Since gene expression levels are not always consistent with their protein content (Maier et al., 2009), the HPA database (https://www.proteinatlas.org/) was used to evaluate it. The genes that meet all the above tests were selected as the real hub genes.

### Construction and Validation of the Prognostic Risk Model

The least absolute shrinkage and selection operator was used to further sort the prognostic genes while the “glmnet” R package was used to construct the prognostic model. The risk score was calculated as follows: Risk score = Sum (each gene’s expression × corresponding coefficient).

Then, the expression levels of genes with different risk scores were determined using a heatmap. The Kaplan–Meier survival curve was also plotted to evaluate the high- and low-risk groups by the log-rank test. Accuracy of the gene signature was determined by generating the receiver operating characteristic (ROC) curves while validation was done using data from the TCGA cohort. PCA and t-SNE were performed to explore the distribution of different groups using the “stats” or “Rtsne (Maaten, 2014)” R package. Univariate and multivariate Cox regression analyses were carried out among the available variables (age, gender, grade, stage) to determine whether the risk score was an independent prognostic predictor for OS via the R package “survival.”

### Functional Enrichment Analysis

To identify the biological functions and pathways correlated with the risk score signature, GO and KEGG enrichment analyses were in the high-and low-risk groups. Moreover, the infiltrating score of 16 immune cells and the activity of 13 immune-related pathways were calculated using the single-sample gene set enrichment analysis (ssGSEA) in the “gsva” R package. GSEA was also performed for the high-and low-expressed real hub genes in the GSE89563 cohort.

### Statistical Analysis

All statistical analyses were performed using the Perl language and R language. The cut-off criteria for significant comparisons were defined as *p* ≤ 0.05.

## Results

### Data Processing and Screening of Differentially Expressed Genes

**FIGURE 2:**
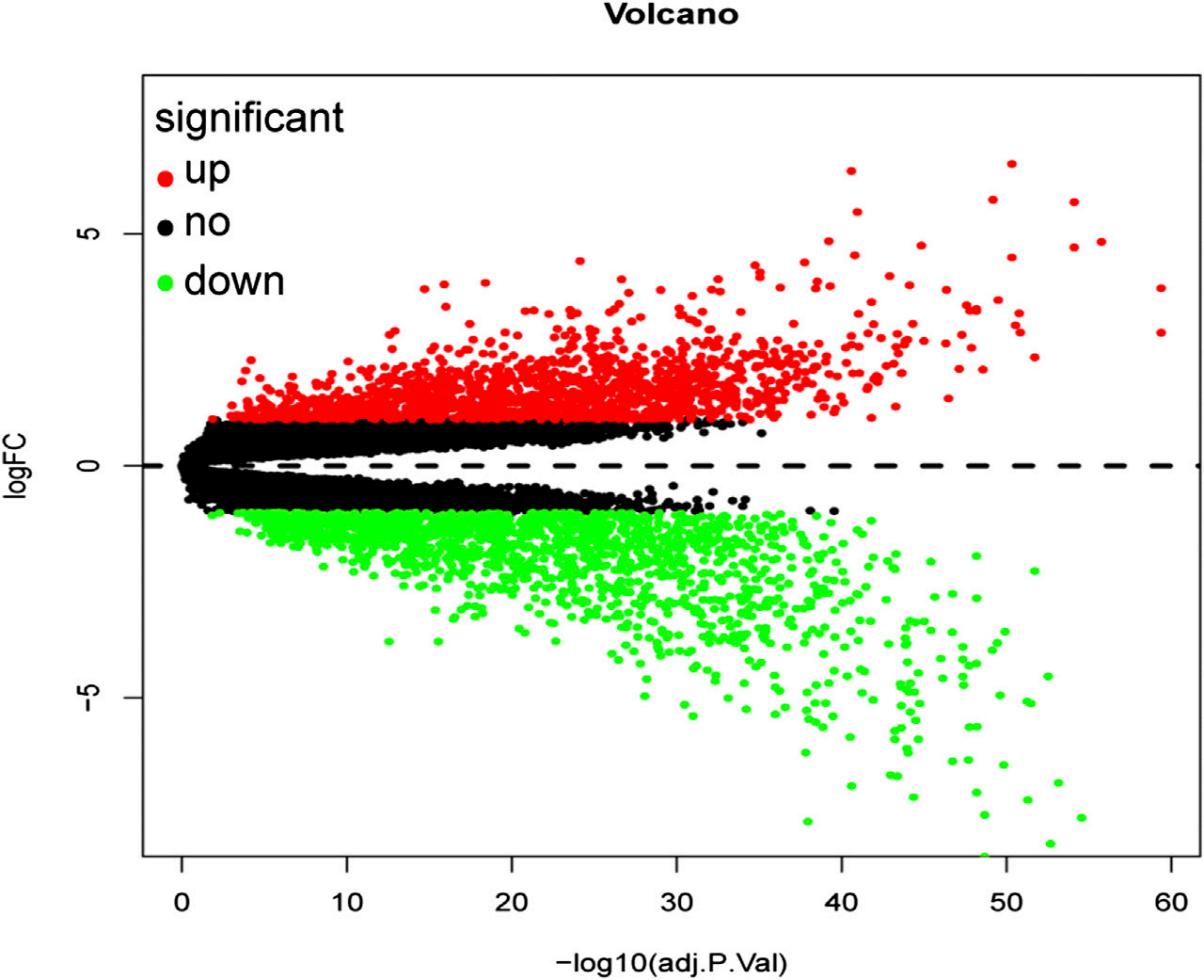
Volcano plot of all differentially expressed genes in GSE53757. A total of 1,579 genes were up-regulated while 1,659 genes were down-regulated. Red: up-regulated DEGs; Black: unchanged DEGs; Green: down-regulated DEGs.

A total of 3,238 DEGs were screened (1,579 up-regulated and 1,659 down-regulated) from a total of 21,655 genes using the FDR <0.05 and log FC (fold change) > 1 threshold. The volcano plot of ccRCC DEGs is presented in Figure 2.

### Weighted Gene Co-Expression Network Construction

**FIGURE 3:**
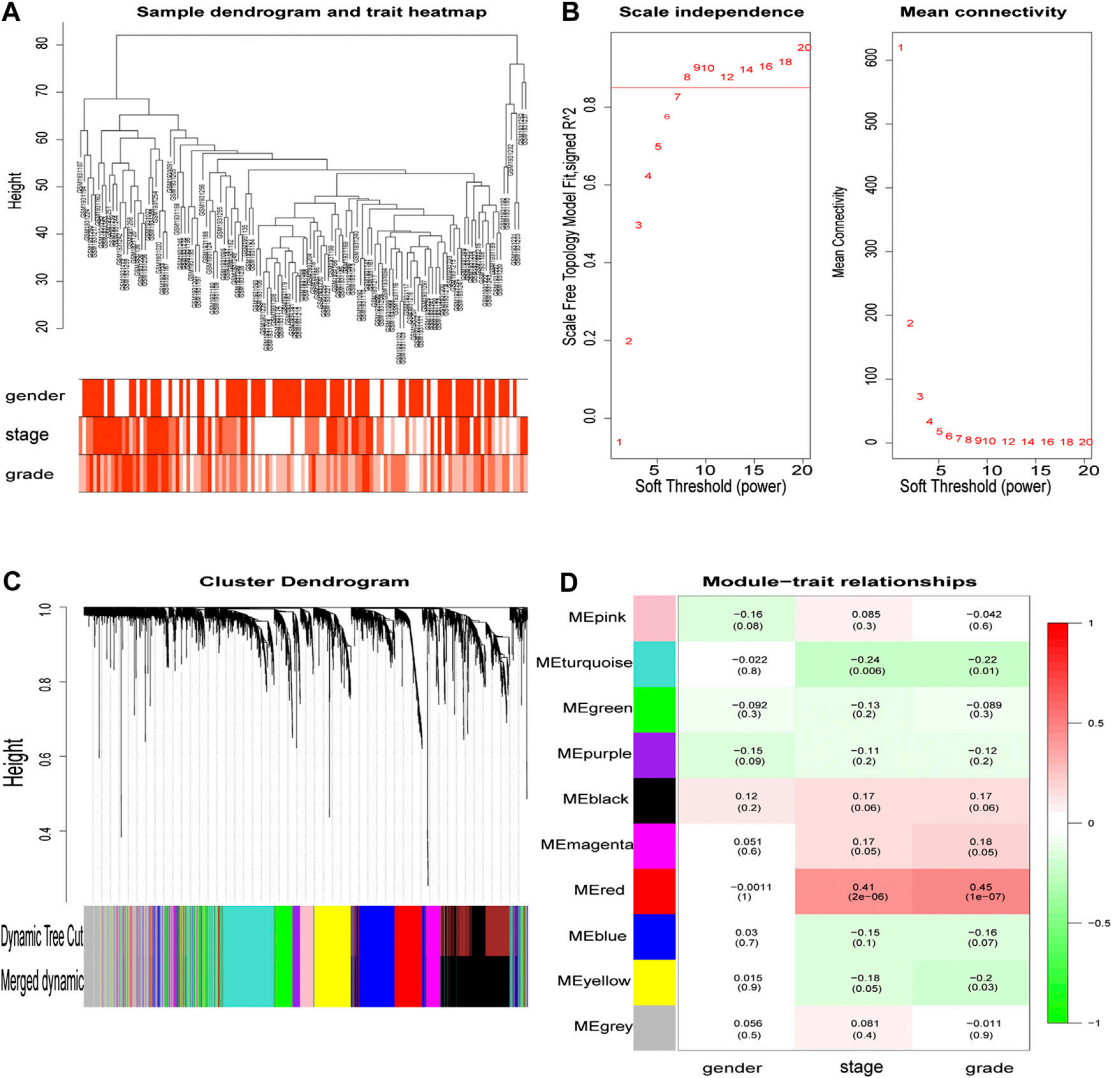
The main steps of WGCNA. Clustering dendrogram of tumor samples with its clinical information. Determination of soft threshold and examination of the scale free topology (β = 8). Hierarchical clustering dendrogram of module eigengenes. Correlation between module and clinical feature, red represents the positive correlation and green represents the negative correlation. The depth of color represents the value of the correlation.

From the hierarchical clustering, there were no outlier samples (Figure 3A). Then, the 3,238 DEGs with similar expression patterns were clustered into modules. β= 8 (scale -free R2 = 0.85) was selected as the soft-thresholding power to ensure a scale-free network (Figure 3B), after which, the network was constructed (Figure 3C). After clustering by dissimilarity between genes, the DEGs were grouped into 11 modules with a minimum size of 50, to establish the gene dendrogram. Given that some modules were similar, a cut-off of 0.25 was made for the module dendrogram. The brown and black modules were combined into a new module, with the color of the new module remaining black. Subsequently, a total of 10 modules were identified.

### Clinically Significant Modules and Their Functions

The correlation value between the gene module’s principal component and the clinical feature was calculated. Figure 3D shows the module that exhibited the highest correlation with the ccRCC clinical stage and pathology (r = 0.41, *p* = 2e-6; r = 0.45, *p* = 1e-7). The red module consisted of 247 genes (195 up-regulated and 52 down-regulated).

## Screening Tests

**FIGURE 4:**
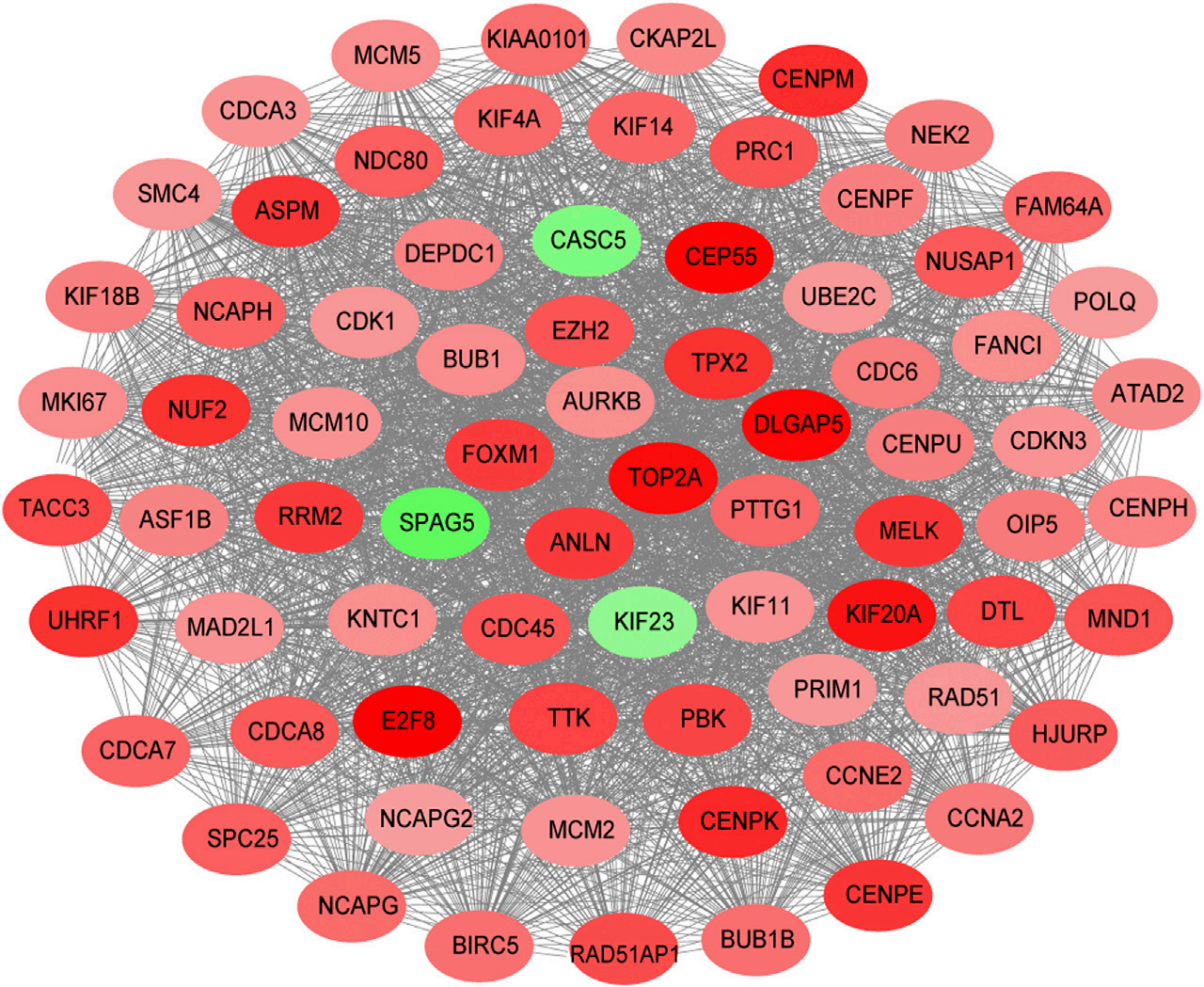
Composition of the molecular complex.

**FIGURE 5:**
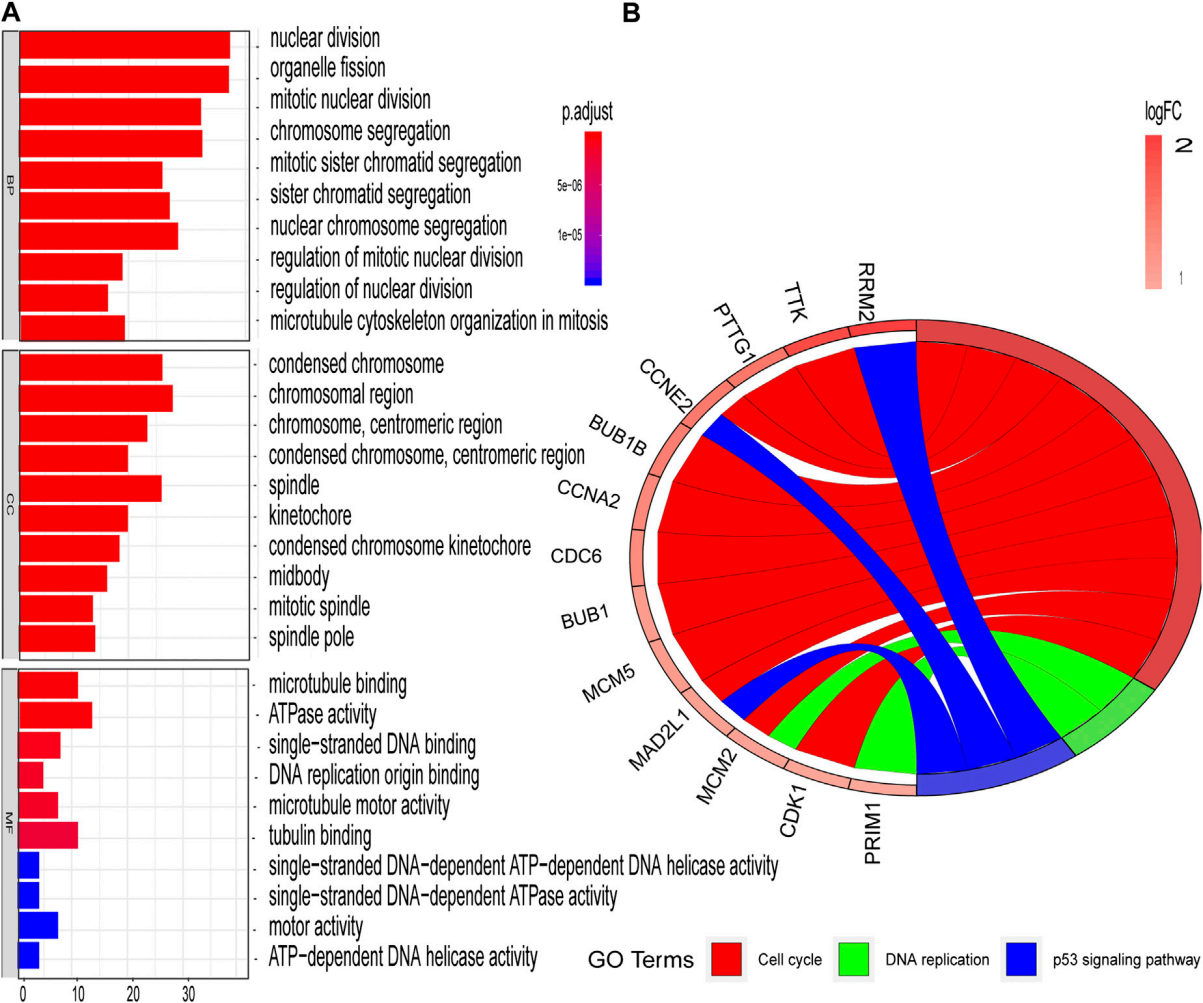
GO and KEGG enrichment analyses of red modules **(A)** Enriched GO terms in Biological processes (BP), Cellular components (CC), and Molecular functions (MF) **(B)** Significantly enriched KEGG pathways.

**FIGURE 6:**
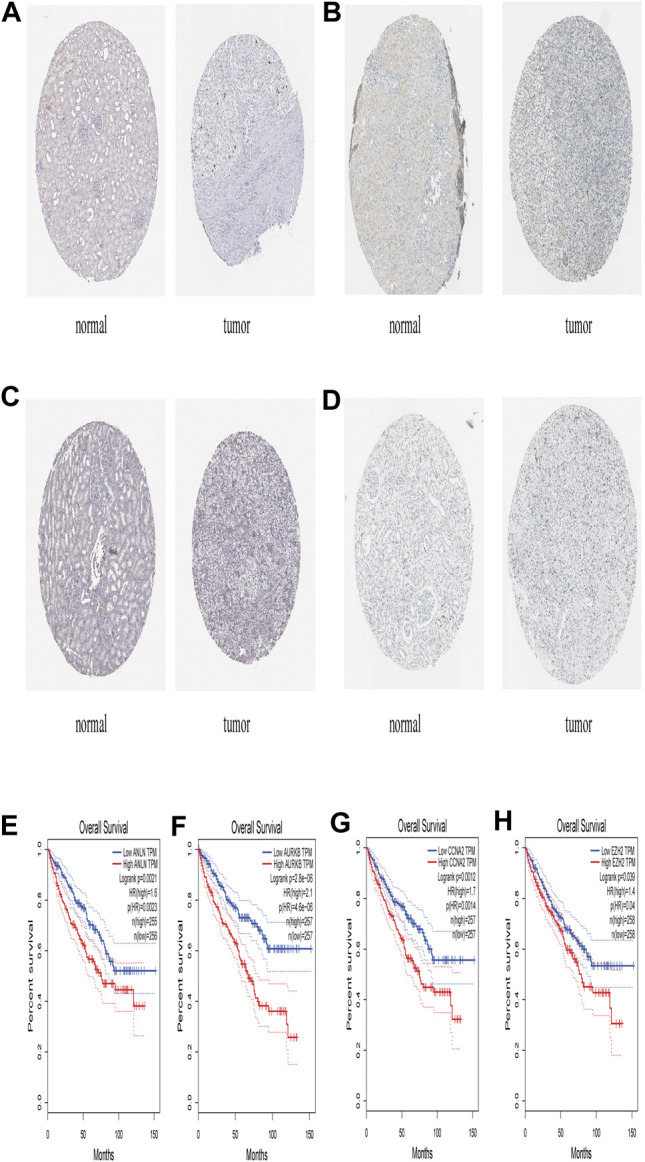
The expression level of ANLN, AURKB, CCNA2, EZH2 in The Human Protein Atlas and its Prognostic value **(A)** Immunohistochemistry results of ANLN in normal tissues (Staining: Low; Intensity: Weak; Quantity: 75–25%; Location: Nuclear) and in ccRCC tissues (Staining: Medium; Intensity: Strong; Quantity: ＜25%; Location: Nuclear) **(B)** Immunohistochemistry results of AURKB in normal tissue (Staining: Not detected; Intensity: Negative; Quantity: None; Location: None) and in ccRCC tissue (Staining: Medium; Intensity: Strong; Quantity: ＜25%; Location: Nuclear) **(C)** Immunohistochemistry results of CCNA2 in normal tissues (Staining: Not detected; Intensity: Negative; Quantity: None; Location: None) and in ccRCC tissues (Staining: Medium; Intensity: Strong; Quantity: ＜25%; Location: Nuclear) **(D)** Immunohistochemistry results of EZH2 in normal tissues (Staining: Not detected; Intensity: Negative; Quantity: None; Location: None) and in ccRCC tissues (Staining: Low; Intensity: Moderate; Quantity: ＜25%; Location: Nuclear) **(E)** Prognostic value of AURKB **(F)** Prognostic value of AURKB **(G)** Prognostic value of CCNA2 **(H)** Prognostic value of EZH2.

The STRING database (https://string-db.org/) was used to construct the PPI in the red module with 228 nodes and 2,910 interactions. Cytoscape and Molecular Complex Detection tool were used to identify the significant. The Molecular complex (Figure 4) presents the most significant hub genes. The red nodes represent the up-regulated genes while the green nodes represent the down-regulated genes. Further, the magnitude of change determined the color depth. Gene interactions were then visualized. Gene Ontology and KEGG pathways in the red module revealed that these genes were mainly involved in “cell cycle,” “DNA replication” and in the “P53 signaling pathway” (Figure 5). The GEPIA database showed that 26 genes were significantly correlated with overall survival while immunohistochemical staining indicated that only 10 genes significantly expressed in the adjacent normal tissues than in cancer tissues (Figure 6).

### Construction and Validation of the Prognostic Risk Model

The LASSO regression analysis was performed to identify the real hub genes with the highest potential prognostic significance. Ultimately, seven genes were retained and used to construct a predictive model. Expression levels of the seven genes and the above determined regression coefficients were used to calculate a risk score for each patient. Risk scores were calculated using the following equation: Risk score = (0.3556 *AURKB) + (0.3660 * FOXM1) + (0.2565 * PTTG1) + (−0.4311 * TOP2A) + (0.0236 * TACC3) + (0.2399 * CCNA2) + (−0.0478 * MELK).

**FIGURE 7:**
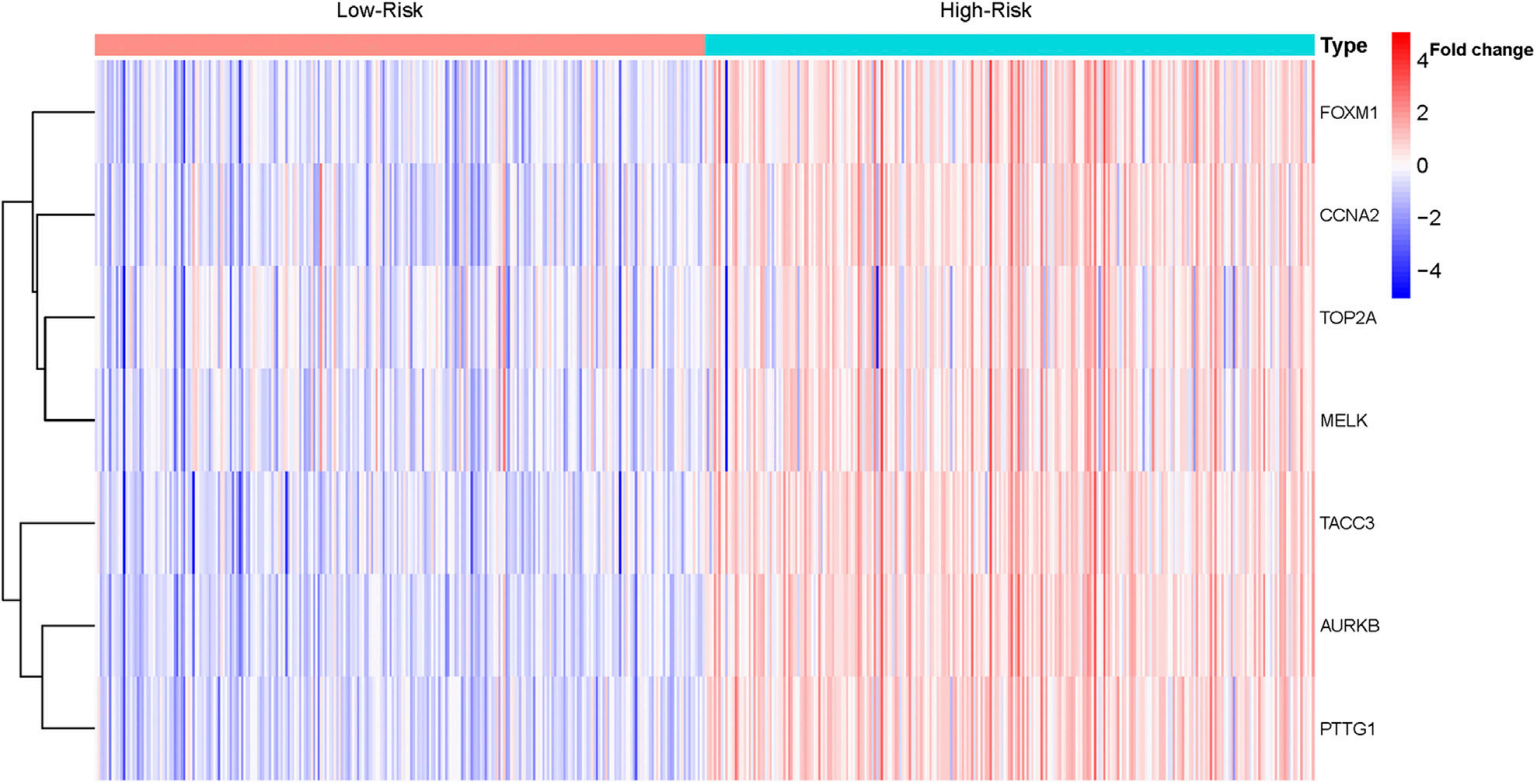
Heatmap of the expression of the seven genes in ccRCC.

**FIGURE 8:**
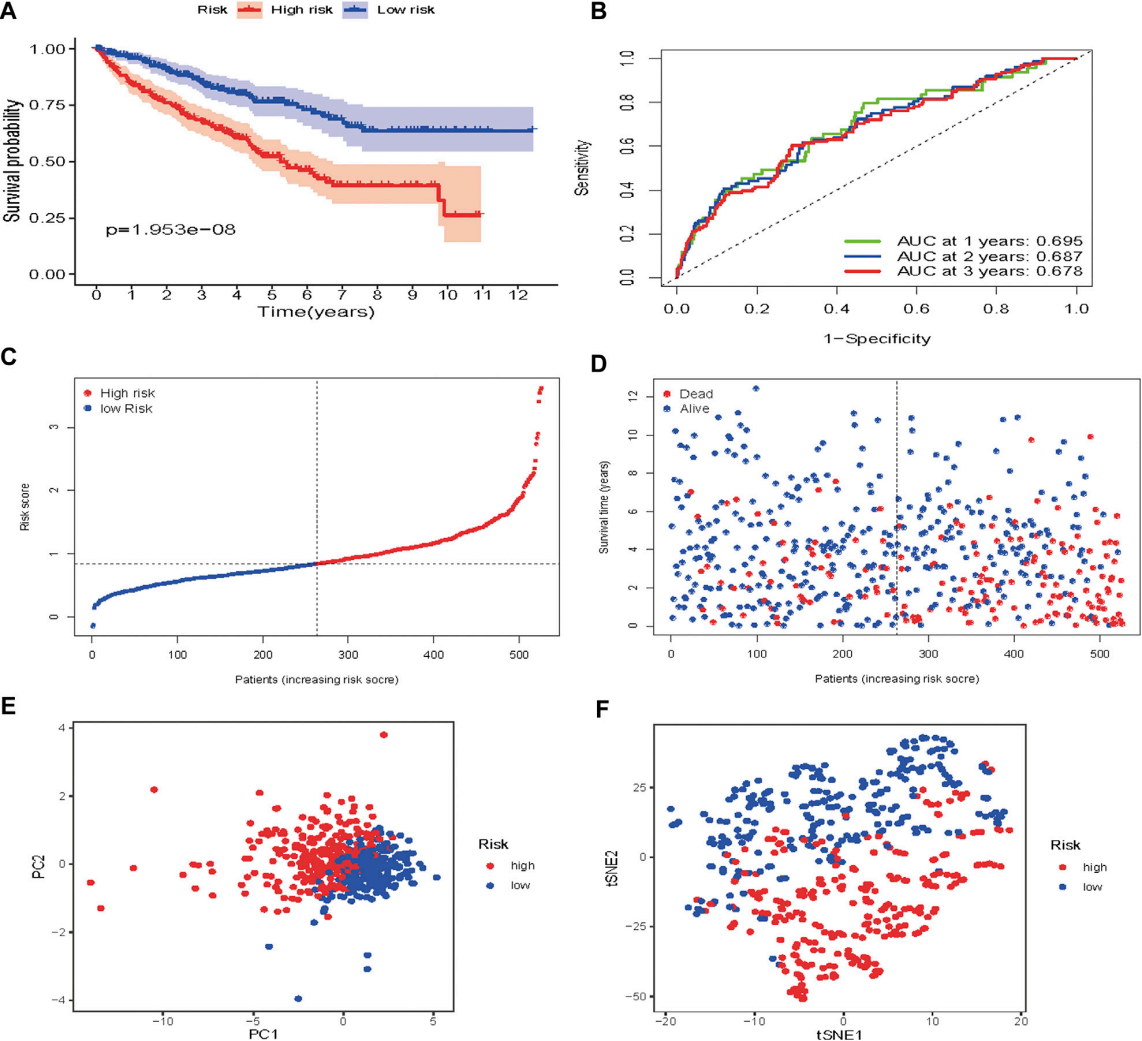
Risk score analysis of the seven-gene prognostic model in TCGA cohort **(A)** Kaplan-Meier curves for the OS of patients in the high-risk group and low-risk group in the TCGA cohort **(B)** AUC of time-dependent ROC curves verified the prognostic performance of the risk score in the TCGA cohort **(C)** Distribution and median value of the risk scores in the TCGA cohort **(D)** Distributions of OS status, OS and risk score in the TCGA cohort **(E)** t-SNE analysis of the TCGA cohort **(F)** PCA plot of the TCGA cohort.

Based on the median risk score, 526 ccRCC patients were assigned into the high-risk (*n* = 263) and low-risk groups (*n* = 263). The heatmap of the expression of 7 genes in the two groups is shown in Figure 7. Low-risk patients exhibited a significantly longer OS compared to the patients in the high-risk group (*p* = 1.953e−08) (Figure 8A). The AUC value for this seven gene risk score signature was 0.695 in the 1 year ROC curve, 0.687 in the 3 years ROC curve, and 0.678 in the 5 years ROC curve (Figure 8B). The risk scores and survival status for each patient in the two subgroups are presented in Figures 8C,D. PCA and t-SNE analysis indicated the patients in different risk groups were distributed in two directions (Figures 8E,F). Univariate analysis revealed that stage and risk score were adverse prognostic factors for survival (Supplementary Figure S1). More interesting, after correction for other confounding factors, multivariable survival analysis remained that risk score was an independent prognostic factor influencing patients with ccRCC (Supplementary Figure S2).

**FIGURE 9:**
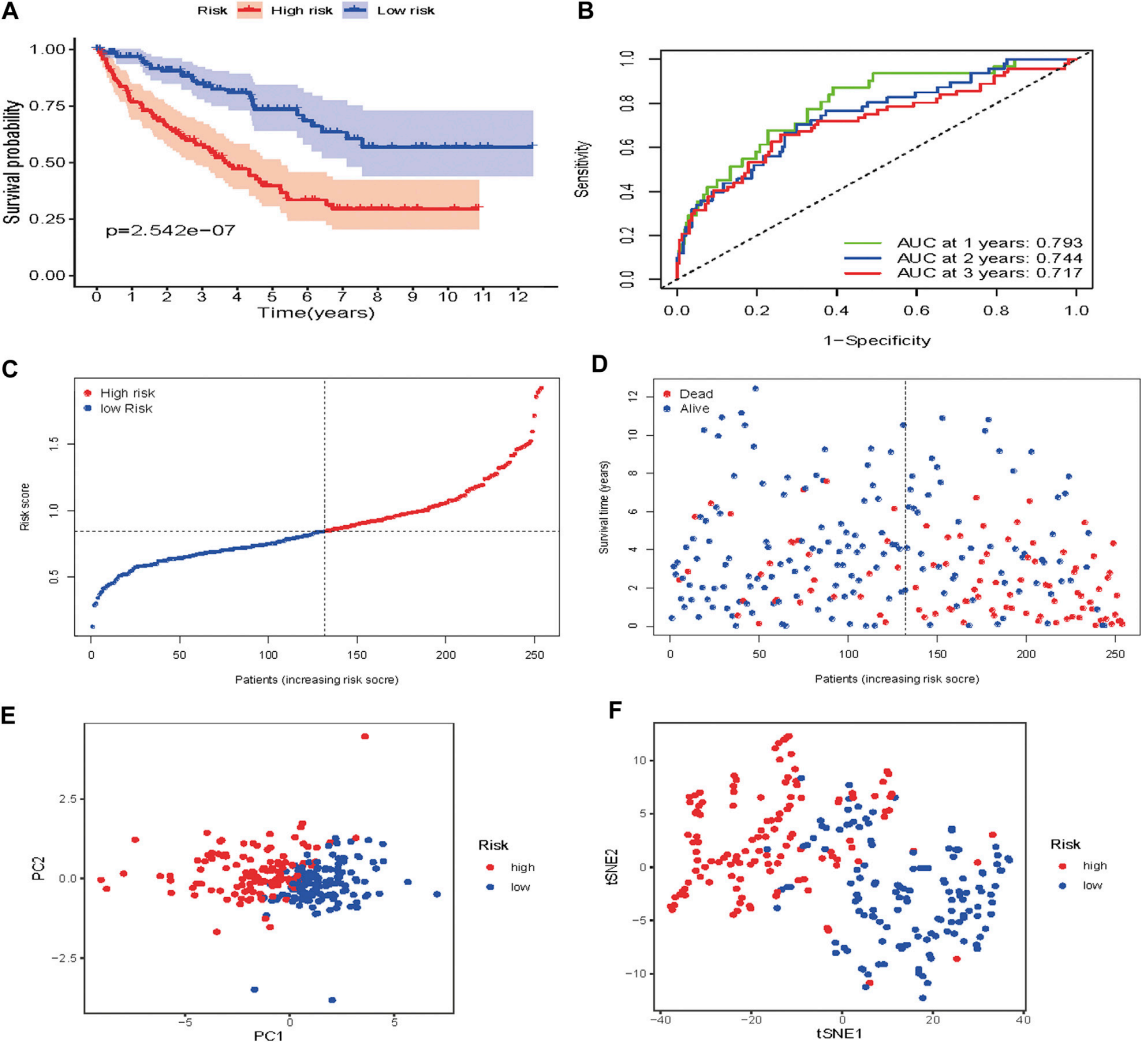
Risk score analysis of the seven-gene prognostic model in the validation cohort **(A)** Kaplan-Meier curves for the OS of patients in the high-risk group and low-risk group **(B)** AUC of time-dependent ROC curves verified the prognostic performance of the risk score model **(C)** Distribution and median value of the risk scores **(D)**Distributions of OS status, OS and risk score in the validation cohort **(E)** t-SNE analysis of the validation cohort **(F)** PCA plot of in the validation cohort.

To verify the prognostic performance of this model, 254 cases were randomly selected from the TCGA database, and their risk scores calculated. Using the TCGA cut-off value, it was found that patients with high-risk scores (*n* = 132) exhibited worse OS than those in the low-risk group (*n* = 122) (*p* = 2.542e−07) (Figure 9A). The AUC value was 0.793 at 1 year, 0.744 at 3 years, and 0.717 at 5 years (Figure 9B). The risk scores and survival status for each patient are shown in Figures 9C,D. PCA and t-SNE analysis results are shown in Figures 9E,F. These results revealed that our prognostic signature had considerable robustness in predicting OS for ccRCC patients.

### 6 Functional Enrichment Analysis

**FIGURE 10:**
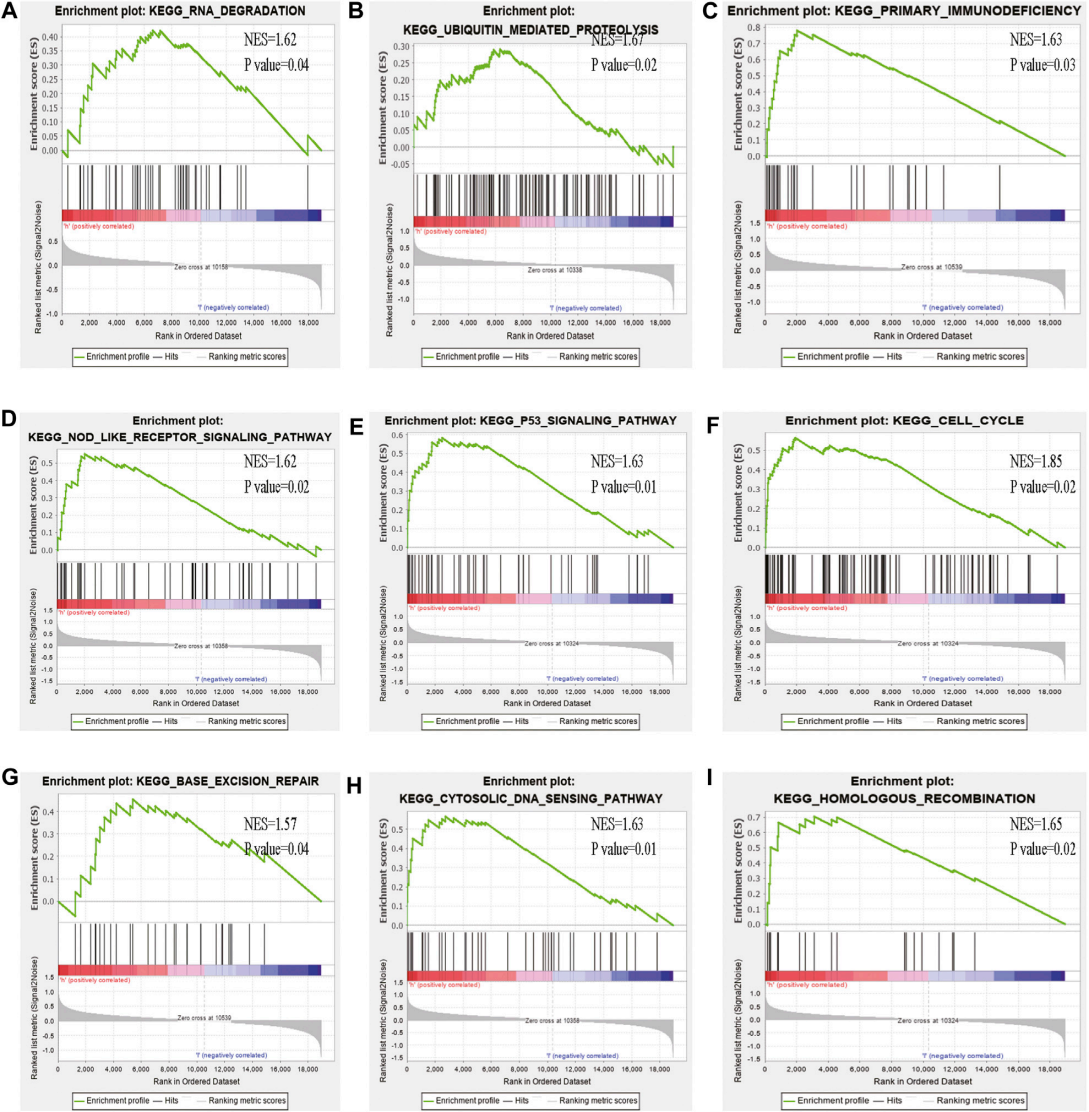
GSEA analysis of the high- and low-risk groups **(A–I)** Some cancer-related pathways were prevalent in the high-risk group: “Nod like receptor signaling pathway,” “P53 signaling pathway,” “cell cycle,” “homologous recombination,” “base excision repair,” “cytosolic dna sensing pathway,” “ubiquitin mediated proteolysis,” “primary immunodeficiency,” “DNA dergradation,” NES, Normalized enrichment score.

**FIGURE 11:**
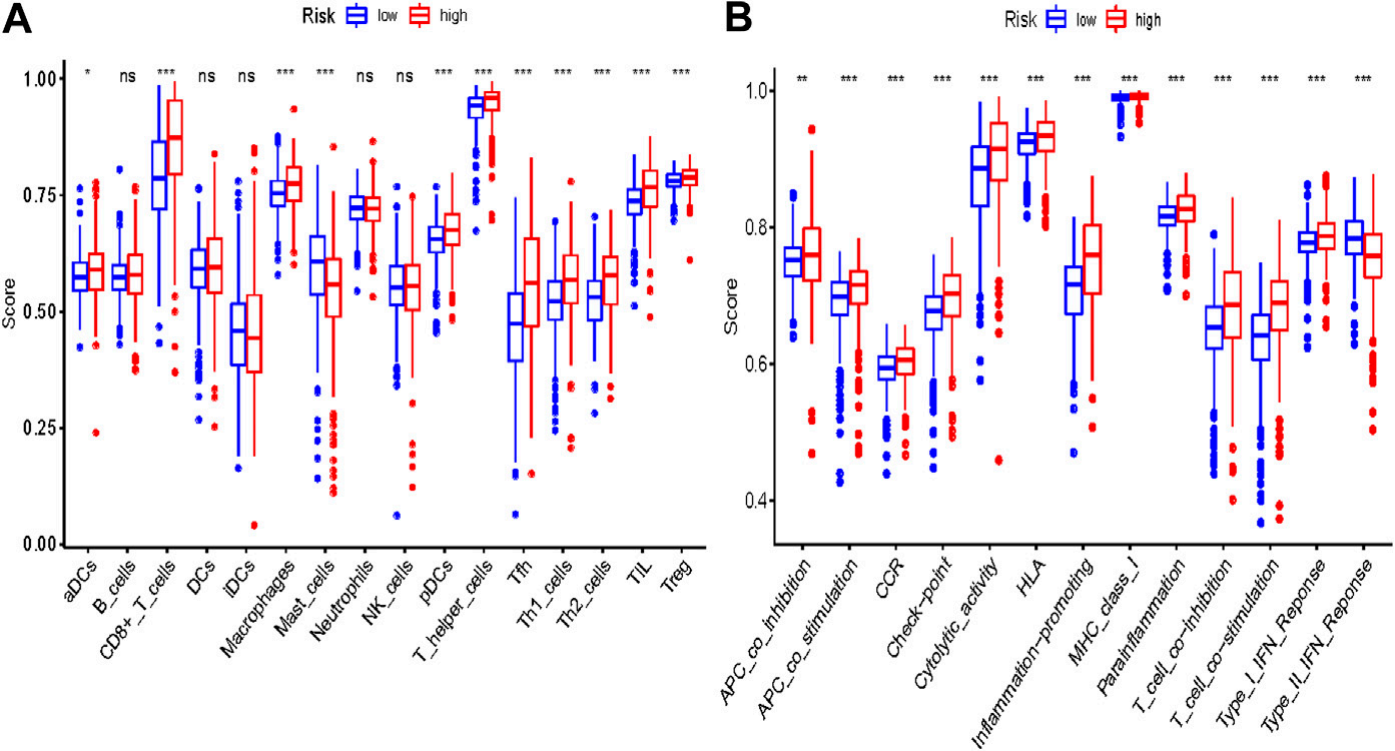
Comparison of the ssGSEA scores between different risk groups in the TCGA cohort. The scores of 16 immune cells **(A)** and 13 immune-related functions **(B)** are displayed in boxplots. Adjusted *p* values were showed as: ns, not significant; *, *p* < 0.05; **, *p* < 0.01; ***, *p* < 0.001.

Some cancer-associated gene sets were found to be significantly elevated in the high-risk score ccRCC patients. These genes were enriched in the P53 signaling pathway, Cell cycle, DNA replication, and Cytosolic DNA-Sensing pathway (Figure 10). To evaluate the correlation between risk score and immune status, we quantified the enrichment scores of diverse immune cell subpopulations, related functions, or pathways using ssGSEA. As shown in Figure 11, the scores for various immune subpopulations were significantly higher in the high-risk group. However, mast cell scores were lower. Fascinatingly, type II IFN response score was low in the high-risk group when compared to the others.

## Discussion

Despite advances in various therapeutic strategies, clinical diagnoses for ccRCC are mostly confirmed in the medium or late stages when mortality and recurrence rates are quite high (Zhao et al., 2018). In precision medicine, this means that more attention should be paid to the dynamic prognosis of disease status. Therefore, we identified a molecular gene complex with significant functions in some cancer-related pathways. Then, overall survival, immunohistochemical, and the least absolute shrinkage selection operator analyses were performed to determine their potential prognostic values. Finally, a risk model that could predict ccRCC prognosis based on six RBP genes was established. The accuracy of this prognostic signature was confirmed by the ROC curve while validation was done using another cohort. Gene set enrichment analysis revealed that some cancer-related phenotypes were significantly abundant in the high-risk group.

Among the seven genes, AURKB and PTTG1 have been reported to act as oncogenes (perezdecastro 2006) during spindle formation or chromosome segregation. Lin Bao et al. showed that AURKB was overexpressed in ccRCC while AURKB knockdown significantly inhibited the migration and invasion of ACHN cells (Bao et al., 2020). Atsushi Okato et al. documented that dual strands of pre-miR-149 act as antitumor miRNAs by targeting FOXM1 in ccRCC cells (Okato et al., 2017). TOP2A, type IIA topoisomerases, which are DNA topoisomerases, are proven therapeutic targets for anticancer and antibacterial drugs. Clinically successful topoisomerase-targeting anticancer drugs act through topoisomerase poisoning, which leads to replication fork arrest and double-strand break formation (Delgado et al., 2018). Chong Zhang et al. found that lncRNA SNHG3 promotes ccRCC proliferation and migration by upregulating TOP2A (Zhang et al., 2019a). However, the mechanism needs further elucidation. TACC3 is involved in chromosomal alignment, separation, and cytokinesis which is associated with p53-mediated apoptosis (Guo and Liu, 2018). Overexpression of TACC3 is correlated with tumor aggression and poor prognosis in prostate cancer (Li et al., 2017). The same phenomenon has been identified in Renal Cell Carcinoma Cells (Guo and Liu, 2018). The levels of CCNA2 are elevated in a variety of tumors such as breast (Gao et al., 2014), cervical (Huo et al., 2019), and liver cancers (Yang et al., 2016). Studies have documented that the oncogenic effect of MELK in ccRCC is exerted through the phosphorylation of PRAS40, an inhibitory subunit of mTORC1, and by disrupting the interaction between PRAS40 and raptor (Zhang et al., 2019b). Given the importance of these seven genes in different cancer types, they might be potential prognostic biomarkers for ccRCC patients. However, studies should be performed to elucidate on their molecular mechanisms.

GSEA analysis showed that some cancer-associated pathways such as P53 pathway, Cell cycle, DNA replication, and Cytosolic DNA-Sensing pathway were enriched in high-risk ccRCC patients. These molecular pathways are involved in carcinogenesis. P53 as a tumor suppressor protein. Inhibition of the p53 pathway may promote ccRCC cell proliferation and inhibit apoptosis (Noon et al., 2010). Through cell cycle and HIF-2α regulation, Notch3 promotes the proliferation of renal cancer cells (Han et al., 2020). DNA sensing activates innate immune responses not only in immune cells such as dendritic cells (DCs) but also in non-immune cells such as fibroblasts and tumor cells. Disorders in DNA sensing may lead to cancer (Han et al., 2020; Qiao et al., 2017).

To inform disease prognosis and progression, several multigene prognostic models have been developed to predict survival for ccRCC patient. Sheng et al. developed an immune-related prognostic model in ccRCC (Shen et al., 2020), while Xiang et al. developed an associated prognostic model for ccRCC based on RNA binding protein expression (Xiang et al., 2020). Construction of a prognostic model for ccRCC using a series cohort and WGCNA methods has not yet been reported. To the best of our knowledge, this is the first study to develop a prognostic model for ccRCC patients using specific types of genes.

Based on our risk score signature, there was a significant difference in OS between high-and low-risk subgroups. Low-risk patients exhibited better survival outcomes than high-risk patients. The ROC curves showed that our prognostic model had good accuracy. The 1, 3 and 5 years AUC values were greater than 0.65, both in the training and testing set. PCA and t-SNE analysis indicated that our prognostic signature model had considerable robustness in predicting OS for ccRCC patients.

However, this study is associated with some limitations. First, the seven genes signature was built and validated using a public dataset, and has not been validated using our own clinical ccRCC case cohort. Second, most ccRCC patients in the TCGA database were Caucasian, and it is, therefore, not clear whether the model has the same predictive effect in non-Caucasian races. Finally, our study was retrospective in nature. Our findings should be validated by a larger prospective study.

In conclusion, we identified and constructed a promising seven gene prognostic signature to predict the clinical outcomes for ccRCC patients. Moreover, this study elucidates on the prognostic value and biological functions of these genes in ccRCC.

## Data Availability

Publicly available datasets were analyzed in this study. This data can be found here: GEO NCBI.

## References

[B1] Bader G. D. Hogue C. W. (2003). An automated method for finding molecular complexes in large protein interaction networks. BMC Bioinformatics 4, 2. 10.1186/1471-2105-4-2 12525261 PMC149346

[B2] Banumathy G. Cairns P. (2010). Signaling pathways in renal cell carcinoma. Cancer Biol. Ther. 10, 658–664. 10.4161/cbt.10.7.13247 20814228 PMC3093809

[B3] Bao L. Zhao Y. Liu C. Cao Q. Huang Y. Chen K. (2020). The identification of key gene expression signature and biological pathways in metastatic renal cell carcinoma. J. Cancer 11, 1712–1726. 10.7150/jca.38379 32194783 PMC7052876

[B32] Delgado J. L. Hsieh C. M. Chan N. L. Hiasa H. (2018). Topoisomerases as anticancer targets. Biochem. J. 475 (2), 373–398. 10.1042/BCJ20160583 29363591 PMC6110615

[B4] Gao T. Han Y. Yu L. Ao S. Li Z. Ji J. (2014). CCNA2 is a prognostic biomarker for ER + breast cancer and tamoxifen resistance. PLoS One 9, e91771. 10.1371/journal.pone.0091771 24622579 PMC3951414

[B5] Gautier L. Cope L. Bolstad B. M. Irizarry R. A. (2004). Affy-analysis of Affymetrix GeneChip data at the probe level. Bioinformatics 20, 307–315. 10.1093/bioinformatics/btg405 14960456

[B6] Guo F. Liu Y. (2018). Knockdown of TACC3 inhibits the proliferation and invasion of human renal cell carcinoma cells. Oncol. Res. 26, 183–189. 10.3727/096504017X14837020772250 28109075 PMC7844598

[B7] Han Q. Han F. Fan Y. Lian B. Xiao J. Sun W. (2020). Notch3 is involved in the proliferation of renal cancer cells via regulation of cell cycle progression and HIF-2α. Oncol. Lett. 20, 1. 10.3892/ol.2020.12242 33154777 PMC7608028

[B8] Horvath S. Zhang B. Carlson M. Lu K. V. Zhu S. Felciano R. M. (2006). Analysis of oncogenic signaling networks in glioblastoma identifies ASPM as a molecular target. Proc. Natl. Acad. Sci. 103, 17402. 10.1073/pnas.0608396103 17090670 PMC1635024

[B9] Huo X. Sun H. Cao D. Yang J. Peng P. Yu M. (2019). Identification of prognosis markers for endometrial cancer by integrated analysis of DNA methylation and RNA-Seq data. Sci. Rep. 9, 9924. 10.1038/s41598-019-46195-8 31289358 PMC6617448

[B10] Lai J.-S. Beaumont J. L. Diaz J. Khan S. Cella D. (2016). Validation of a short questionnaire to measure symptoms and functional limitations associated with hand-foot syndrome and mucositis in patients with metastatic renal cell carcinoma. Cancer 122, 287–295. 10.1002/cncr.29655 26457466

[B11] Li Q. Ye L. Guo W. Wang M. Huang S. Peng X. (2017). Overexpression of TACC3 is correlated with tumor aggressiveness and poor prognosis in prostate cancer. Biochem. Biophysical Res. Commun. 486, 872–878. 10.1016/j.bbrc.2017.03.090 28336437

[B12] Maaten L. V. D. (2014). Accelerating t-SNE using tree-based algorithms, JMLR 15, 3221–3245. 10.5555/2627435.2697068

[B13] Maier T. Güell M. Serrano L. (2009). Correlation of mRNA and protein in complex biological samples. FEBS Lett. 583, 3966–3973. 10.1016/j.febslet.2009.10.036 19850042

[B14] Miller J. A. Horvath S. Geschwind D. H. (2010). Divergence of human and mouse brain transcriptome highlights Alzheimer disease pathways. Proc. Natl. Acad. Sci. USA. 107, 12698–12703. 10.1073/pnas.0914257107 20616000 PMC2906579

[B15] Moch H. Cubilla A. L. Humphrey P. A. Reuter V. E. Ulbright T. M. (2016). The 2016 WHO classification of tumours of the urinary system and male genital organs-Part A: renal, penile, and testicular tumours. Eur. Urol. 70, 93–105. 10.1016/j.eururo.2016.02.029 26935559

[B16] Noon A. P. Vlatković N. Polański R. Maguire M. Shawki H. Parsons K. (2010). p53 and MDM2 in renal cell carcinoma. Cancer 116, 780–790. 10.1002/cncr.24841 20052733 PMC3536467

[B17] Okato A. Arai T. Yamada Y. Sugawara S. Koshizuka K. Fujimura L. (2017). Dual strands of pre-miR-149 inhibit cancer cell migration and invasion through targeting FOXM1 in renal cell carcinoma. Ijms 18, 1969. 10.3390/ijms18091969 PMC5618618 28902136

[B18] Qiao J. Tang H. Fu Y.-X. (2017). DNA sensing and immune responses in cancer therapy. Curr. Opin. Immunol. 45, 16–20. 10.1016/j.coi.2016.12.005 28088707

[B19] Ritchie M. E. Phipson B. Wu D. Hu Y. Law C. W. Shi W. (2015). Limma powers differential expression analyses for RNA-sequencing and microarray studies. Nucleic Acids Res. 43, e47. 10.1093/nar/gkv007 25605792 PMC4402510

[B20] Shen C. Liu J. Wang J. Zhong X. Dong D. Yang X. (2020). Development and validation of a prognostic immune-associated gene signature in clear cell renal cell carcinoma. Int. Immunopharmacol. 81, 106274. 10.1016/j.intimp.2020.106274 32044664

[B21] Su G. Morris J. H. Demchak B. Bader G. D. (2014). Biological network exploration with Cytoscape 3. Curr. Protoc. Bioinformatics 47, 8 13 11-24. 10.1002/0471250953.bi0813s47 25199793 PMC4174321

[B22] Sun G. Li Y. Peng Y. Lu D. Zhang F. Cui X. (2019). Identification of differentially expressed genes and biological characteristics of colorectal cancer by integrated bioinformatics analysis. J. Cell Physiol. 234, 15215. 10.1002/jcp.28163 30652311

[B23] Suttle A. B. Ball H. A. Molimard M. Hutson T. E. Carpenter C. Rajagopalan D. (2014). Relationships between pazopanib exposure and clinical safety and efficacy in patients with advanced renal cell carcinoma. Br. J. Cancer 111, 1909–1916. 10.1038/bjc.2014.503 25349968 PMC4229638

[B24] Voineagu I. Wang X. Johnston P. Lowe J. K. Tian Y. Horvath S. (2011). Transcriptomic analysis of autistic brain reveals convergent molecular pathology. Nature 474, 380–384. 10.1038/nature10110 21614001 PMC3607626

[B25] Xiang Y. Zhou S. Jian H. Zhong C. Ma Q. Sun Z. (2020). Development and validation of a prognostic model for kidney renal clear cell carcinoma based on RNA binding protein expression. Aging 12, 25356–25372. 10.18632/aging.104137 33229623 PMC7803486

[B26] Yan X. Wan H. Hao X. Lan T. Li W. Xu L. (2019). Importance of gene expression signatures in pancreatic cancer prognosis and the establishment of a prediction model. Cmar, 11, 273–283. 10.2147/CMAR.S185205 PMC6312063 30643453

[B27] Yang F. Gong J. Wang G. Chen P. Yang L. Wang Z. (2016). Waltonitone inhibits proliferation of hepatoma cells and tumorigenesis via FXR-miR-22-CCNA2 signaling pathway. Oncotarget 7, 75165–75175. 10.18632/oncotarget.12614 27738335 PMC5342731

[B28] Zhang B. Horvath S. (2005). A general framework for weighted gene co-expression network analysis. Stat. Appl. Genet. Mol. Biol. 4, 17. 10.2202/1544-6115.1128 16646834

[B29] Zhang C. Qu Y. Xiao H. Xiao W. Liu J. Gao Y. (2019a). LncRNA SNHG3 promotes clear cell renal cell carcinoma proliferation and migration by upregulating TOP2A. Exp. Cell Res. 384, 111595. 10.1016/j.yexcr.2019.111595 31505165

[B30] Zhang H. Wei P. Lv W. Han X. Yang J. Qin S. (2019b). MELK is pregulated in advanced clear cell renal cell carcinoma and promotes disease progression by phosphorylating PRAS40. Cell Transpl. 28, 37S–50S. 10.1177/0963689719890860 PMC7016465 31813279

[B31] Zhao H. Cao Y. Wang Y. Zhang L. Chen C. Wang Y. (2018). Dynamic prognostic model for kidney renal clear cell carcinoma (KIRC) patients by combining clinical and genetic information. Sci. Rep. 8, 17613. 10.1038/s41598-018-35981-5 30514856 PMC6279814

