# Supplementary figures and images for: Development and Validation of a Prognostic Gene Signature in Clear Cell Renal Cell Carcinoma
Source: Front Mol Biosci. 2021 Apr 8;8:609865. doi: 10.3389/fmolb.2021.609865 (PMC8098777; doi:10.3389/fmolb.2021.609865)

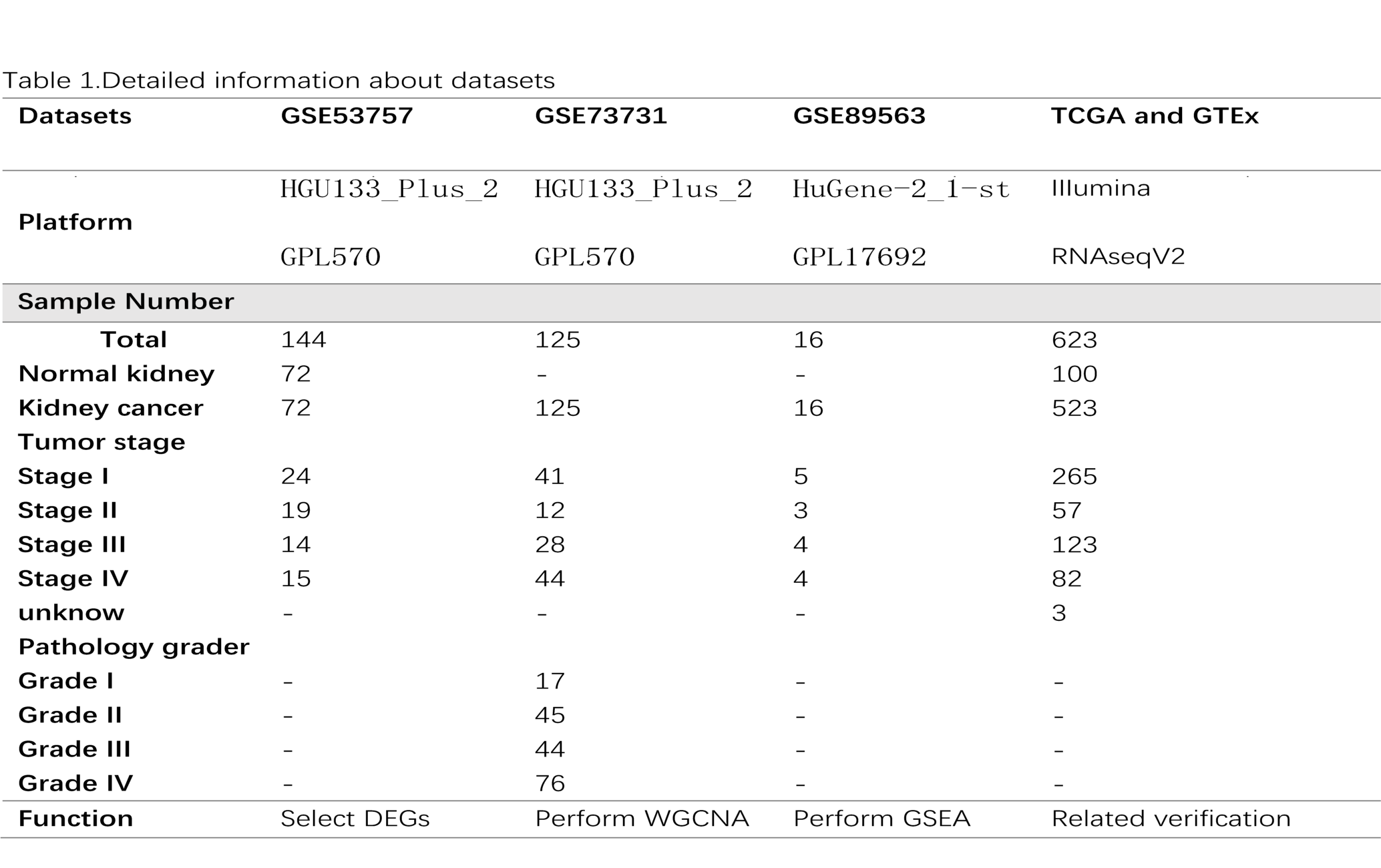

Supplement: Supplementary file 1 [file image1.tif]
