# Supplementary material for: Development and Validation of a Prognostic Gene Signature in Clear Cell Renal Cell Carcinoma
Source: Front Mol Biosci. 2021 Apr 8;8:609865. doi: 10.3389/fmolb.2021.609865 (PMC8098777; doi:10.3389/fmolb.2021.609865)
Supplement: Supplementary file 2 [file image2.tif]

Frontiers | Development and validation of a prognostic gene
signature in clear cell renal cell carcinoma | Molecular Biosciences


- About
- Journals
- Research Topics
- Articles
- More

Submit

My Frontiers

Office

- TSOF
  - TSOF
  - Article Production

Typesetter 3

frontiersproduction@tnq.co.in

- Profile
- Settings & Privacy
- Help Center
- Logout

Submit

**Impact Factor 4.188** | **CiteScore 5.1**More on impact ›

|  |  |
| --- | --- |
| Frontiers in Molecular Biosciences | Molecular Diagnostics and Therapeutics |

Toggle navigation


Section


- (current)Section
- About
- Articles
- Research topics
- For authors 
  - Why submit?
  - Fees
  - Article types
  - Author guidelines
  - Review guidelines
  - Submission checklist
  - Contact editorial office
  - Submit your manuscript
- Editorial board

- *Article alerts*

Articles


**Suggest a Research Topic >**

- 85
  total views

 View Article Impact

**Suggest a Research Topic >**

##### SHARE ON

- Facebook

  0
- Twitter

  0
- LinkedIn

  0
- AddThis

  New


## Original Research ARTICLE

Front. Mol. Biosci.
| doi: 10.3389/fmolb.2021.609865

# Development and validation of a prognostic gene signature in clear cell renal cell carcinoma Provisionally accepted The final, formatted version of the article will be published soon. **Notify me**

Zhan Chuanchuan1 and Bai Peiming1\*

- 1Zhongshan Hospital, Xiamen University, China

Clear cell renal cell carcinoma (ccRCC), one of the most common urologic cancer types, has a relatively good prognosis. However, the confirmed patients are mostly in the medium or late stages, when the mortality and recurrence rates are quite high. It’s necessary to conduct real-time information tracking, dynamic prognosis analysis for these patients. Hence, we downloaded the RNA-seq and corresponding clinical information of ccRCC from The Cancer Genome Atlas (TCGA) database and Gene Expression Omnibus (GEO) database. and 3238 differentially expressed genes were identified between normal and ccRCC tissues. Through series of Weighted Gene Co-expression Network, overall survival, immunohistochemical and the least absolute shrinkage selection operator (LASSO) analyses, seven prognosis-related genes (AURKB, FOXM1, PTTG1, TOP2A, TACC3, CCNA2, and MELK) were screened out and its risk score signature was constructed. Survival analysis indicated that high-risk scores had significantly worse overall survival than low-risk patients. The good accuracy of this prognostic signature was confirmed by the ROC curve analysis and was further validated in another cohort. Gene set enrichment analysis demonstrated some cancer-related phenotypes were significantly gathered in the high-risk group. Overall, our results demonstrate that this risk model could potentially improve individualized diagnostic and therapeutic strategies.

Keywords: 
Kidney cancer, Microarray, WGCNA, Targeting therapy, novel markers, prognostic model

Received: 17 Oct 2020;
Accepted: 19 Jan 2021.

Copyright: © 2021 Chuanchuan and Peiming. This is an open-access article distributed under the terms of the Creative Commons Attribution License (CC BY). The use, distribution or reproduction in other forums is permitted, provided the original author(s) and the copyright owner(s) are credited and that the original publication in this journal is cited, in accordance with accepted academic practice. No use, distribution or reproduction is permitted which does not comply with these terms.

\* Correspondence: 
PhD. Bai Peiming, Zhongshan Hospital, Xiamen University, Xiamen, China, baipeiming@xmu.edu.cn

Write a comment...

Add

##### COMMENTARY

##### ORIGINAL ARTICLE

##### People also looked at

## Risk Signature Related to Immunotherapy Reaction of Hepatocellular Carcinoma Based on the Immune-Related Genes Associated With CD8+ T Cell Infiltration

Yiping Zou, Zhihong Chen, Hongwei Han, Shiye Ruan, Liang Jin, Yuanpeng Zhang, Zhengrong Chen, Zuyi Ma, Qi Lou, Ning Shi and Haosheng Jin

## Spatio-temporal Inversion using the Selection Kalman Model

Maxime Conjard and Henning Omre

## Single-Cell Analysis Reveals Characterization of Infiltrating T Cells in Moderately Differentiated Colorectal Cancer

Xi Yang, Quan Qi, Yuefen Pan, Qing Zhou, Yinhang Wu, Jing Zhuang, Jiamin Xu, Mingyue Pan and Shuwen Han

## Transcriptomic Analysis of Glycolysis-Related Genes Reveals an Independent Signature of Bladder Carcinoma

Zezhong Mou, Chen Yang, Zheyu Zhang, Siqi Wu, Chenyang Xu, Zhang Cheng, Xiyu Dai, Xinan Chen, Yuxi Ou and Haowen Jiang

**Suggest a Research Topic >**

×

#### Supplementary Material

  

There is no supplementary material currently available for this article

Loading supplemental data...

  

|  | File Name |  |
| --- | --- | --- |
|  | Image 1.TIF |  |
|  | Image 2.TIF |  |
|  | Image 3.TIF |  |

  

Close

- About Frontiers
- Institutional Membership
- Books
- News
- Frontiers' social media
- Contact
- Careers
- Submit
- Newsletter
- Help Center
- Terms & Conditions
- Privacy Policy

© 2007 - 2021 Frontiers Media S.A. All Rights Reserved

### Privacy Preference Center

Our website uses cookies that are necessary for its operation. Additional cookies are only used with your consent. These cookies are used to store and access information such as the characteristics of your device as well as certain personal data (IP address, navigation usage, geolocation data) and we process them to analyse the traffic on our website in order to provide you a better user experience, evaluate the efficiency of our communications and to personalise content to your interests. Some cookies are placed by third-party companies with which we work to deliver relevant ads on social media and the internet. Click on the different categories' headings to change your cookie preferences. Click on "More Information" if you wish to learn more about how data is collected and shared.
More information

### Manage Consent Preferences

#### Strictly Necessary Cookies

Always Active

These cookies are necessary for the website to function and cannot be switched off in our systems. They are usually only set in response to actions made by you which amount to a request for services, such as setting your privacy preferences, logging in or filling in forms. You can set your browser to block or alert you about these cookies, but some parts of the site will not then work. These cookies do not store any personally identifiable information.

#### Analytics Cookies

Analytics Cookies

These cookies allow us to count visits and traffic sources so we can measure and improve the performance of our site. They help us analyse which pages are the most and least popular and see how visitors move around the site.    All information these cookies collect is aggregated and therefore anonymous.

#### Functional Cookies

Functional Cookies

These cookies enable the website to provide enhanced functionality and personalisation. They may be set by us or by third party providers whose services we have added to our pages. If you do not allow these cookies then some or all of these services may not function properly.

#### Advertising Cookies

Advertising Cookies

These cookies may be set through our site by our advertising partners. They may be used by those companies to build a profile of your interests and show you relevant adverts on other sites.    They do not store directly personal information, but are based on uniquely identifying your browser and internet device. If you do not allow these cookies, you will experience less targeted advertising.

### Back Button Performance Cookies

Vendor Search  Search Icon

Filter Icon

Clear

checkbox label label

Apply Cancel

Consent Leg.Interest

checkbox label label

checkbox label label

checkbox label label

Confirm My Choices
